# A Numerical Investigation of Risk Factors Affecting Lumbar Spine Injuries Using a Detailed Lumbar Model

**DOI:** 10.1155/2018/8626102

**Published:** 2018-04-17

**Authors:** Jiajia Zheng, Liang Tang, Jingwen Hu

**Affiliations:** ^1^School of Technology, Beijing Forestry University, Beijing 100083, China; ^2^University of Michigan Transportation Research Institute, Ann Arbor, MI 48109, USA

## Abstract

Recent field data showed that lumbar spine fractures occurred more frequently in late model vehicles than early ones in frontal crashes. However, the lumbar spine designs of the current crash test dummies are not accurate in human anatomy and have not been validated against any human/cadaver impact responses. In addition, the lumbar spines of finite element (FE) human models, including GHBMC and THUMS, have never been validated previously against cadaver tests. Therefore, this study developed a detailed FE lumbar spine model and validated it against cadaveric tests. To investigate the mechanism of lumbar spine injury in frontal crashes, effects of changing the coefficient of friction (COF), impact velocity, cushion thickness and stiffness, and cushion angle on the risk of lumbar spine injuries were analyzed based on a Taguchi array of design of experiments. The results showed that impact velocity is the most important factor in determining the risk of lumbar spine fracture (*P* = 0.009). After controlling the impact velocity, increases in the cushion thickness can effectively reduce the risk of lumbar spine fracture (*P* = 0.039).

## 1. Introduction

Safety designs in newer cars generally provided better protections to occupants than those in older cars. However, several recent field data analyses have shown that lumbar spine fractures occurred more frequently in late model vehicles than the early ones in frontal crashes [1, 2].

This increasing trend of lumbar spine fractures in frontal crashes is extremely concerning, because none of the current crash test programs, including FMVSS 208, US-NCAP, and IIHS, considered lumbar spine injury in their safety evaluation process. This is partially due to the fact that none of the current crash test dummies can accurately estimate lumbar spine fracture risks. Current crash test dummies were designed to focus on estimating injury risks to the head, neck, chest, and lower extremities, and their lumbar spine designs were not based on real human anatomy and were not validated against any human/cadaver impact responses. As a result, although lumbar spine loadings could be measured in a crash test, they may not necessarily reflect the real injury mechanism or injury risk in frontal crashes. In addition, lumbar spines of the available FE human models, such as GHBMC (Global Human Body Model Consortium) model and THUMS (Total Human Model for Safety) model, have never been validated previously against cadaver tests. More recently, Arun et al. [3] applied the stiffness values obtained from cadaver tests directly to the lumbar spine of the GHBMC simplified model. However, it is a rigid body-based lumbar spine model. It is lacking of detailed anatomical structures of the lumbar spine and cannot estimate strain and stress in the vertebrae.

Factors affecting the lumbar spine fracture have been discussed extensively in the literature. Results indicated that older occupants (65+ years) were five times more likely to sustain spinal injury compared to younger occupants [4], women had more lumbar spine fractures than men [5], and lower bone quality is associated with an increased number of lumbar spine fractures within the CIREN cases analyzed [6]. Lumbar spine posture was also found to be an important factor affecting lumbar spine injuries. It was reported that slouched posture may cause an increase in stress on the lumbar vertebrae [7] and more reclined postures are associated with a higher lumbar vertebrae fracture risk by reconstructing real-world motor vehicle crashes [8]. Crash pulse magnitude and shape are also crucial for determining lumbar spine injury risk. For example, cadaver tests under axial loading showed that triangular pulses produced pelvic fractures with no lumbar spine fractures, while a sigmoid-shaped pulse produced no pelvic fractures but did produce an L1 burst fracture [9]. In addition, more severe crash pulses may lead to higher lumbar spine injury risks [10].

The objectives of the present study were to develop a detailed FE lumbar spine model, validate it against cadaveric tests, and use it to investigate the effects of changing the coefficient of friction (COF), impact velocity, cushion thickness and stiffness, and cushion angle on the risk of lumbar spine injuries. This study could provide better understanding of how to design countermeasures to reduce occupant lumbar spine injuries in the new generation of vehicle models.

## 2. Lumbar Spine Model Development and Validation

### 2.1. Model Development

**Figure 1:**
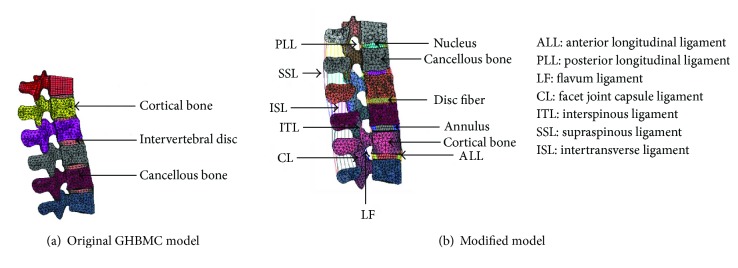
Comparison between original and modified lumbar spine models.

**Figure 2:**
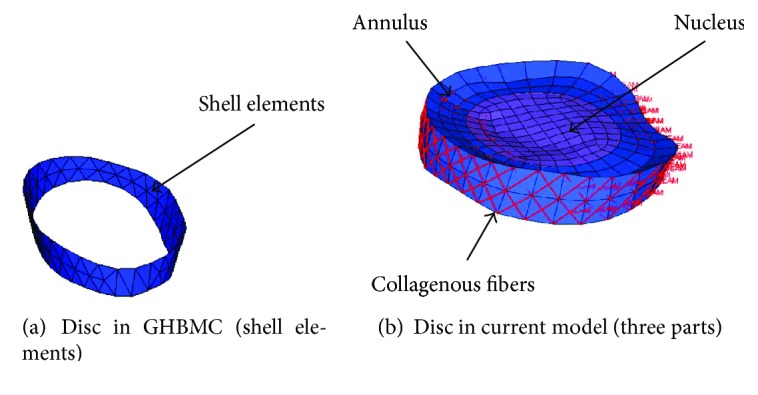
Comparison of the discs between GHBMC and modified lumbar models.

In this study, the GHBMC model was selected as the baseline model to be modified due to its accurate geometrical representation. In the original GHBMC lumbar spine model (Figure 1(a)), vertebrae (T12-L5) were represented by a rigid material, intervertebral discs were modeled by shell elements (Figure 2(a)), and no ligaments were modeled. In the modified model (Figure 1(b)), the intervertebral disc was separated into three parts including nucleus, annulus, and collagenous fibers (Figure 2(b)). A 0.5 mm layer of shell element was added to the outside of the original vertebrae, representing the cortical bone. Detailed ligaments modeled by beam elements were added between the vertebrae. Nucleus, annulus, and cancellous bones were modeled by solid elements. Collagenous fibers and ligaments were modeled by beam elements. The facet joint articulations were simulated as the tiebreak contact between adjacent endplates. Note that muscles were ignored in the current study.

The modified lumbar model (Figure 1(b)) was composed of 64,338 elements and 12,295 nodes. The cancellous bones and cortical bones were modeled by MAT_SIMPLIFIED_JOHNSON_COOK in LS_DYNA [11, 12] with 1.3% and 0.94% effective plastic failure strains, respectively. The endplates of the discs were modeled by an elastic material, and the endplates of vertebrae were modeled by MAT_SIMPLIFIED_JOHNSON_COOK. The nucleus and annulus were modeled by MAT_MOONEY-RIVLIN_RUBBER [11, 12]. Accordingly, the shear modulus constant *A* of the nucleus and annulus were 0.64 MPa and 0.24 MPa, and the constant *B* of nucleus and annulus were −0.16 MPa and −0.06 MPa, respectively [12]. A linear elastic material was used for ligaments. Selections of stiffness of ligaments (Table 1) were based on a previous study [13]. The collagenous fibers were represented by a nonlinear load-displacement curve obtained from the literature [14]. Because external lamellae are stiffer than internal lamellae, the fibers in different layers were weighted according to a previous research [15]. To tune the model responses, the material properties of the bones and ligaments were adjusted slightly to match the test results during model validations. The material properties of all the parts in the modified lumbar model are listed in Table 2.

### 2.2. Validation against Nonfailure Tests

**Figure 3:**
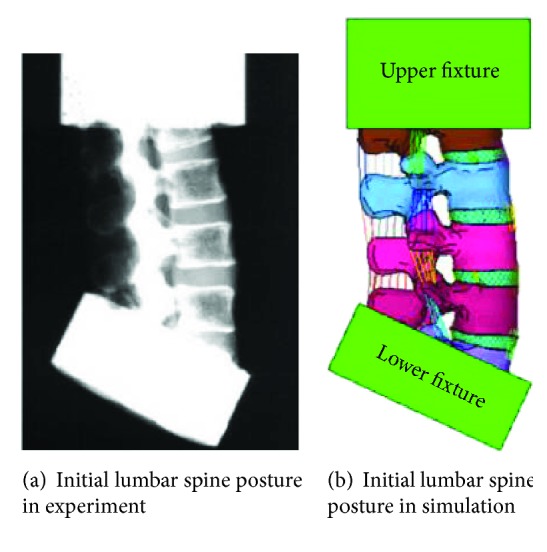
Initial posture of the lumbar spine of cadaver experiment and simulation.

Cadaveric tests from Demetropoulos et al. [17] were used to validate the modified lumbar model. It is important to adjust the initial posture of the lumbar spine to be consistent with the experimental data before validation [18]. Therefore, a presimulation was conducted to adjust the spine curvature to match the specimen pretest condition, as shown in Figure 3. Impact simulations were performed at different loading modes corresponding to the testing conditions. As a result, six simulations under compression, anterior shear, posterior shear, lateral shear, extension, and lateral bending were performed with the modified lumbar spine model. Each simulation was run at a displacement rate of 100 mm/sec. The maximum displacements were set to be the same as those in the tests. During these simulations, T12 was fixed to the fixture on the top, and L5 was attached to the fixture at the bottom. In the compression and shear conditions, load was applied from the lower fixture with the upper fixture rigidly constrained. In the bending condition, displacement was applied from the lower fixture, and a bending moment was applied to the superior end of the lumbar spine by a cable. Force, moment, and angular displacement were measured at the upper fixture.

**Figure 4:**
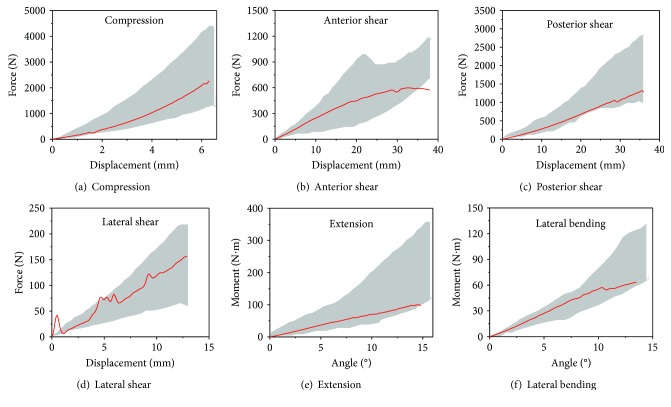
Response comparisons between tests and simulations (red: simulation results, grey: test corridors).

Figure 4 shows load-displacement curves in each loading condition for the modified lumbar spine model. In compression, posterior shear, lateral shear, extension and lateral bending, simulation results shown in red line fell within test corridors reasonably well.

### 2.3. Validation against Failure Tests

**Figure 5:**
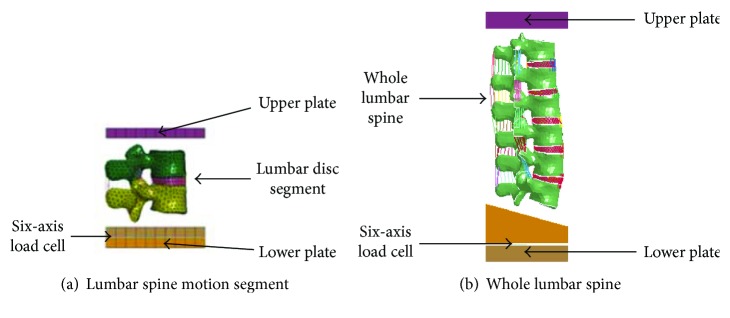
Test configurations for dynamic compression with failure.

Cadaver tests with tissue failure conducted by Duma et al. [19] were used to validate the modified lumbar model. The validation simulations were set up under the same configurations as those in the dynamic compression tests (Figure 5). The first simulation used a lumbar spine motion segment (Figure 5(a)), and the second simulation used the entire lumbar spine (Figure 5(b)). During the simulations, the superior end of the lumbar spine was attached to the upper plate, and the inferior end was attached to the lower plate. A prescribed motion was applied on the upper plate while the lower plate was fixed. All the failure simulations were loaded at 1.0 m/s. Force and moment were calculated in these simulations under different loadings.

**Figure 6:**
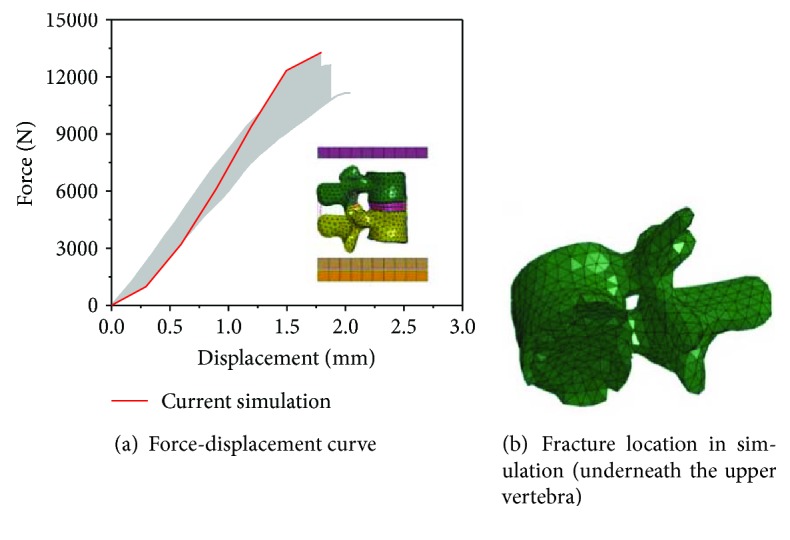
Lumbar spine motion segment test and simulation results under compression.

**Figure 7:**
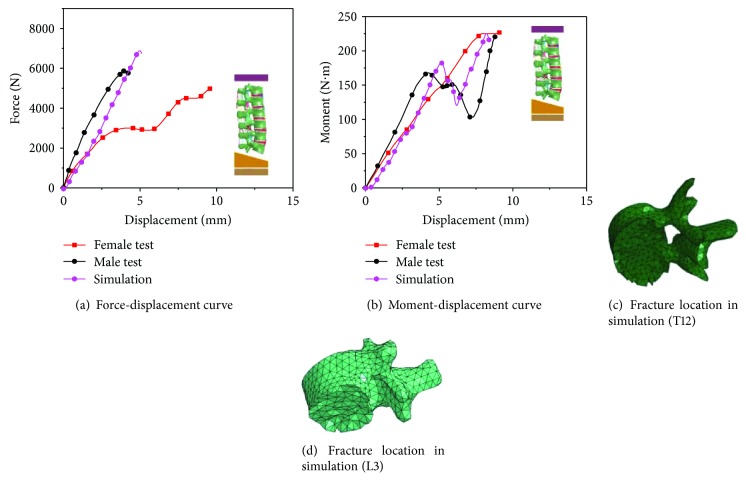
Test and simulation results of whole lumbar spines.

As shown in Figure 6, the model-predicted force-displacement curve for the motion segment configuration fell within the experimental corridor. Good correlations between the simulation and the test for the entire lumbar spine compression were also achieved, as shown in Figure 7. In addition, the locations of fractures in the simulation were consistent with those in the test (underneath the T12 and L3), as shown in Figures 7(c) and 7(d).

## 3. Design of Experiment Analysis

A DoE analysis based on Taguchi Array was used to study the effects of multiple factors on the risk of lumbar spine injuries. In this study, 5 factors were investigated, including cushion stiffness, cushion thickness, cushion angle, the coefficient of friction, and impact velocity.

**Figure 8:**
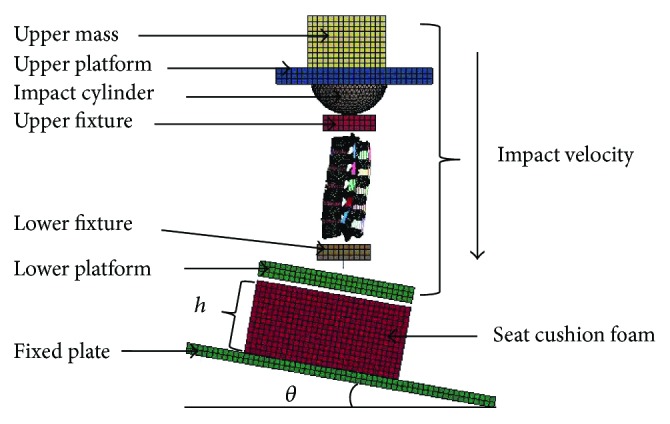
Setup used in Taguchi design of experiment analysis.

The setup of the simulations in the DoE analysis is shown in Figure 8 [20]. The upper mass was attached to the upper platform to simulate the mass of the torso, head-neck, and upper extremities. The interaction between the upper platform and the upper fixture was in a form of a laterally oriented cylinder. The lumbar spine was fixed at cranial and caudal ends to the upper fixture and lower fixture, respectively [20]. A cushion foam, of which the thickness was defined as *h*, was attached to the fixed plate as shown in Figure 8, and its density was varied for the DoE analysis. The fixed plate was constrained at a cushion angle of *θ*. The initial impact velocity was applied to the upper mass, upper platform, and impact cylinder, through the upper fixture, lumbar spine specimen, and lower fixture, and finally reached the lower platform, cushion foam, and the fixed plate (Figure 8).

The initial setup conditions of the impact velocity, cushion angle, thickness, and density were 0.1 m/s, 0 degree, 20 mm, and 20 kg/m^3^, respectively. The coefficient of friction (COF) between the lower platform and the seat cushion was ranged from 0 to 0.9. Four different levels were assigned for each factor (Table 3). Because most lumbar injuries were reported as vertebral fractures, the maximum principal strain in the bony structures was selected to evaluate the risk of lumbar fracture [21]. These setups resulted in a total of 16 simulations based on the Taguchi Array, as shown in Table 4. One-way analysis of variance (ANOVA) and analysis of covariance (ANCONA) were performed using SPSS 20.0.

**Figure 9:**
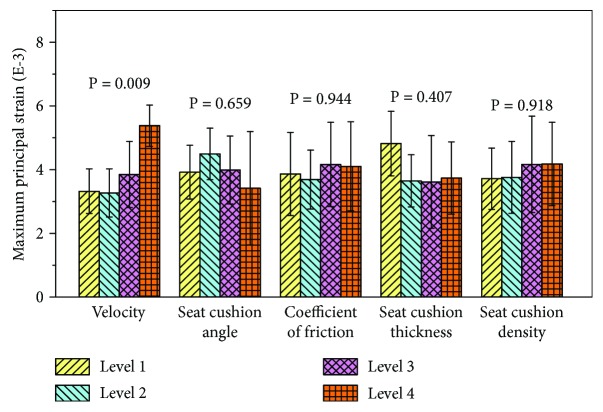
The average maximum principal strain with respect to all 5 factors simulated using the Taguchi array.

The average values of the maximum principal strains of different factor categories are shown in Figure 9. Impact velocity (*P* = 0.009) had the most significant influence on the risk of lumbar injuries. With impact velocity varying between 0.1 and 0.5 m/s, the maximum principal strain fluctuated between 0.0032 and 0.0038, but it increased rapidly to 0.0053 when impact velocity rose to 0.7 m/s. Except the impact velocity, no other factor was statistically significant (*P* > 0.05).

**Figure 10:**
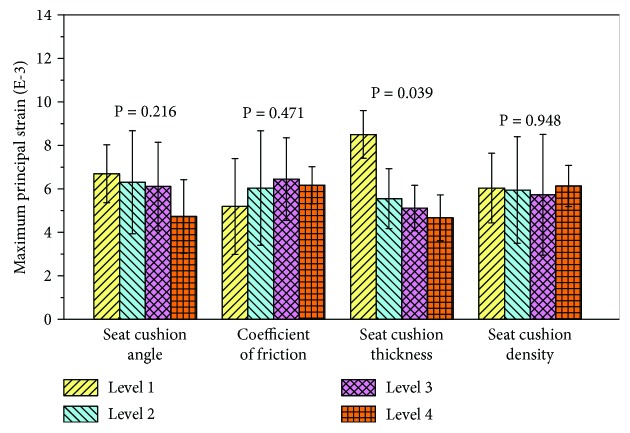
The average maximum principal strain with respect to the left 4 factors without impact velocity (impact velocity was 0.7 m/s).

Because the impact velocity dominated the lumbar fracture risk in the Taguchi array, a one-way ANOVA was then conducted for the remaining 4 factors by controlling the impact velocity as a constant. The average values of the maximum principal strains of different factor categories are shown in Figure 10. The cushion thickness (*P* = 0.039) became significant for the lumbar injuries. An increase in the cushion thickness will lead to more energy absorption and in turn lower lumbar spine fracture risk. Even though other factors were not significant, some general trends have also been noted. For example, with an increase in the COF, the lumbar spine fracture risk generally increased. For the cushion angle, the highest injury risk occurred when the lumbar spine orientation was perpendicular to the cushion. These trends are widely consistent to the findings from other studies on lumbar spine injuries [22] and cervical spine injuries [23].

## 4. Conclusions

A detailed modified FE lumbar spine model based on the GHBMC model was developed and validated against available cadaveric tests which appeared in the literature. Risk factors affecting lumbar spine injuries were investigated using the modified model. Results of the DoE analysis demonstrated that the impact velocity is a significant factor influencing the lumbar injuries. After controlling the impact velocity, cushion thickness is another significant factor influencing lumbar injuries. An increase of cushion thickness or decrease of cushion stiffness will reduce the lumbar spine fracture risk. Additionally, minimizing the COF between the padding and the lumbar spine can reduce the lumbar spine injury risk.

One of the limitations of this study is that human factors such as BMI (body mass index), sex, and age were not considered. Older occupants have higher risk of lumbar spine fractures, and their lumbar spine curvatures may be different to the younger adults [24]. In addition, muscles were not included in the current lumbar model. Future studies may investigate the effects of human factors and muscle activations on the lumbar spine injury risks using parametric human models [25, 26].

## Figures and Tables

**Table 1:** Stiffness of ligaments used in the current study (unit: N/mm).

Ligaments	T12-L1	L1-L2	L2-L3	L3-L4	L4-L5	L5-S1
ALL	32.9	32.4	20.8	39.5	40.5	32.9
PLL	10	17.1	36.6	10.6	25.8	10
LF	24.2	23	25.1	34.5	27.2	24.2
CL	31.7	42.5	33.9	32.3	30.6	31.7
ISL	12.1	10	9.6	18.1	8.7	12.1
SSL	15.1	23	24.8	34.8	18	15.1
ITL	15.1	23	24.8	34.8	18	15.1

**Table 2:** Material properties used in the current study.

Segments	MAT	*R*0 (t/mm^3^)	*E* (MPa)/*K* (N/mm)	Poisson ratio	*A* ^∗^ (MPa)	*B* ^∗^ (MPa)	*N*	*C*	PSFAIL	SIGMAX (MPa)
Cortical bone	98	1.83*E* − 09	11,740	0.3	106.7	100	0.1	1	9.40*E* − 03	150
Cancellous bone	98	1.80*E* − 10	259	0.25	1.71	20	1	1	1.30*E* − 02	1.985
Endplate vertebra	98	1.06*E* − 09	9450	0.3	5.67	100	1	3	1.89*E* − 02	7.1
Endplate disc	1	1.20*E* − 09	200	0.3						
Disc annulus	27	1.20*E* − 09	0.49		0.24	−0.06				
Disc nucleus	27	1.00*E* − 09	0.495		0.64	−0.16				
Fiber	71	1.20*E* − 09	Curves							
Ligaments	74	1.00*E* − 09								

^∗^In LS_DYNA material keywords, the strain energy density of MAT_MOONEY-RIVLIN_RUBBER is defined as a function of constant *A*, constant *B*, and Poisson ratio [16].

**Table 3:** Magnitude of different levels for the five factors selected for DOE analysis.

Level	Velocity (m/s)	Cushion angle (degrees)	Cushion thickness (mm)	Cushion density (kg/m^3^)	Coefficient of friction
1	0.1	0	20	20	0
2	0.3	10	60	30	0.3
3	0.5	20	100	40	0.6
4	0.7	30	140	50	0.9

**Table 4:** Maximum principal strain of 16 simulations in DOE analysis.

Number	Velocity (mm/ms)	Cushion angle (°)	Coefficient of friction	Cushion thickness (mm)	Cushion density (kg/m^3^)	Maximum principal strain (E-3)
1	0.5	10	0.9	140	20	4.08
2	0.5	20	0.6	20	40	5.17
3	0.7	20	0.3	60	20	4.6
4	0.1	20	0.9	100	30	2.97
5	0.5	30	0	60	30	2.72
6	0.5	0	0.3	100	50	3.43
7	0.3	20	0	140	50	3.22
8	0.1	30	0.3	140	40	2.51
9	0.7	0	0.6	140	30	5.14
10	0.1	0	0	20	20	3.82
11	0.1	10	0.6	60	50	3.99
12	0.7	30	0.9	20	50	6.08
13	0.3	10	0.3	20	30	4.21
14	0.3	0	0.9	60	40	3.29
15	0.3	30	0.6	100	20	2.36
16	0.7	10	0	100	40	5.7
